# A socioecological description of the influencing factors to midwives’ management of preeclampsia in a Ghanaian tertiary hospital

**DOI:** 10.1371/journal.pone.0291036

**Published:** 2023-09-13

**Authors:** Isabella Garti, Michelle Gray, Angela Bromley, Benjamin (Jing-Yu) Tan

**Affiliations:** 1 Faculty of Health, Charles Darwin University, Darwin, Australia; 2 School of Nursing and Midwifery, Edith Cowan University, Joondalup, Australia; University of Ghana Medical School, GHANA

## Abstract

**Introduction:**

In low-resource settings, midwives are the first contact for women with preeclampsia and lead the coordination of care. Unfavourable preeclampsia outcomes create a burden for women, families, and the health system. It is therefore important to understand the unique context of midwives’ practice and the complex factors that influence the delivery of maternal healthcare.

**Aim:**

This qualitative study explored the perspectives of key stakeholders in a tertiary hospital in Ghana regarding the facilitators and barriers influencing midwives’ provision of preeclampsia care using a socioecological model.

**Methods:**

Semi-structured interviews were conducted with 42 participants comprising senior managers (n = 7) and hospital midwives (n = 35) in 2021. Thematic analysis used Braun and Clarke’s six-step method, and the findings were organised within four levels of the socioecological model: individual, interpersonal, organisational, and public policy.

**Results:**

Two main themes were identified: 1) Facilitators of preeclampsia management, and 2) Barriers to preeclampsia management. Facilitators were identified at three levels (individual, interpersonal, and organisational) and included midwives’ knowledge of preeclampsia; midwives’ self-efficacy; midwives’ skillset to enhance preeclampsia care; collaborative practice; and strategies for preeclampsia care quality improvement. At the individual level, the barriers were inadequate pre-service preparation, lack of evidence-based midwifery care, and colleagues’ work attitudes. Hierarchical decision-making and staff views of women’s risk perceptions were identified as barriers at the interpersonal level. At the organisational level, the barriers were: scarce resources and staff shortages, and a lack of midwifery-specific guidelines. Two barriers were identified within the public policy level: the high cost of preeclampsia care and issues with the referral system.

**Conclusion:**

Multi-faceted factors play a significant role in midwives’ management of preeclampsia. Hence context-specific multi-level interventions have the potential to improve the quality-of-care women in Ghana receive.

## 1. Introduction

Hypertensive disorders of pregnancy (HDP) encompass a range of medical complications characterised by high blood pressure that can occur either before or after 20 weeks of gestation and last for 6 weeks postpartum [1]. Preeclampsia is a common hypertensive disorder and a leading cause of maternal and neonatal morbidity and mortality worldwide [2]. The disorder is more prevalent in low- and middle-income countries compared to high-income countries [3]. Preeclampsia is diagnosed when new onset maternal hypertension is noticed after 20 weeks gestation with proteinuria and other signs of multi-system involvement such as acute kidney injury, liver dysfunction, or neurological features. Preeclampsia can develop suddenly and progress rapidly and mothers may experience lifelong complications like cardiovascular disease post-pregnancy [1, 2]. The baby may be small for gestational age, born preterm, have low Apgar scores, or be stillborn [2]. These complications highlight the urgent need for effective management and monitoring of preeclampsia to minimise maternal and neonatal morbidity and mortality.

The high burden of preeclampsia in low and middle-income counties is challenging because insufficient resources impact the diagnosis and management of this condition [4]. Preeclampsia poses an enormous threat to maternal and fetal survival in low-resource settings like Ghana where the incidence is reported at about 7.6% of all pregnancies [5]. In the past three decades, studies have shown preeclampsia is a leading cause of maternal mortality in Ghanaian tertiary hospitals [5, 6], with adverse outcomes well documented [5, 7, 8]. For example, in the eastern part of Ghana, there is a high prevalence of preeclampsia. Out of 5,609 births in one tertiary hospital, between 2018 and 2020, 314 mothers were diagnosed with preeclampsia. The majority of these mothers were young, nulliparous, and had a lower level of education [9]. Statistics from another study involving five referral hospitals show that out of 447 women, a significant majority 76%) had preeclampsia. Among these mothers, 83% had preterm babies, 14% experienced stillbirth, and 19% faced neonatal mortality [10]. With the current trends, key strategies are required to reduce the burden of preeclampsia in Ghana and consequently reduce maternal and neonatal mortality.

Maternal healthcare in LMICs is impacted by inadequate healthcare infrastructure, drug shortages, and lack of healthcare professionals trained to global standards resulting in inappropriate care at all levels [11, 12]. Prevailing sociocultural, economic, and geographic barriers lead to delayed decision making and limited access to emergency obstetric care, further worsening the likelihood of poor outcomes [11]. Understanding these interrelated factors is essential for comprehensive strategies to improve maternal health. The management of preeclampsia in LMICs is challenging owing to these interrelated factors which impact on the quality of care. Therefore, addressing one factor alone is insufficient and a multi-faceted approach is needed to study, develop and implement tailored strategies and actions that are contextual to improve patient outcomes.

In the Ghanaian context, these factors align with the challenges faced in maternal health. The standard of maternal care is impacted by a lack of qualified medical professionals and scarce resources [13]. In Ghanaian hospitals, insufficient staffing, long wait times, non-adherence to treatment protocols, and the lack of respectful maternity care are known to impact on treatment outcomes and, by extension, maternal health outcomes [13–16]. Despite the availability of free antenatal care and high antenatal coverage in Ghana, women often choose traditional birth attendants for home births, a preference driven by the high costs associated with hospital-based care [17]. The lack of Ghanaian preeclampsia guidelines that reflect current recommendations is reported in another paper which highlights that Ghana lacks consistent implementation of international recommendations for midwives in managing preeclampsia [18]. Also documented are the numerous challenges in the referral system, such as the inaccessibility of health centres and lack of ambulances [19]. Low health literacy of the population has also been linked to poor maternal decision making and poor maternal risk perceptions [20].

Ghana’s maternal mortality ratio is 310 per 100,000 live births [21]. Although the country made substantial progress towards achieving the millennium development goals (MDG’s) through several initiatives such as the free birthing policy, progress has stalled [22], and urgent action is required to turn the tide around in the wake of the Sustainable Development Goals (SDG’s). In Ghana, across all levels of health service delivery, midwives are the main providers of care in the pregnancy-labour continuum [15]. Therefore, they are well placed to deliver lifesaving interventions such as the administration of magnesium sulphate and first-line parenteral antihypertensives. Yet, Ghanaian health facilities at the district level and below suffer from a scarcity of practicing midwives [23]. Many midwives choose to pursue alternative healthcare professions or leave due to limited educational prospects, salary concerns, and reluctance to work in rural areas, leading to a critical shortage of midwives [23, 24]. Although midwifery training integrates the International Confederation of Midwives (ICM) core competencies, continuing professional development (CPD) opportunities for skill maintenance are few.

The existing preeclampsia research conducted in Ghana has primarily concentrated on pathogenesis, trends, outcomes, diagnostic criteria, drug efficacy, and long-term implications thereby, contributing to our understanding of the disorder and providing the evidence base to accelerate diagnosis and treatment [8, 9, 25, 26]. However, there is a paucity of research into midwives’ management of preeclampsia and the only published study identified focused on midwives’ management of eclampsia in two district hospitals. In that investigation, issues faced by midwives at the primary care levels, included understaffing and the lack of access to magnesium sulphate [15]. To date, there is a gap in the literature concerning the facilitators and barriers to preeclampsia management and relatively few studies exist examining the quality of care and underlying factors [16, 27]. This existing body of research focuses on health professionals in general rather than specifically on midwives [27], creating a gap in understanding the unique role and contributions of midwives in preeclampsia management. So far, there has been little discussion about the interacting factors that enhance or negatively impact midwives’ preeclampsia management. This needs a more comprehensive examination. This study is necessary to advance knowledge on the influences of microsystem contexts so that future quality improvement strategies are created holistically for sustained reduction in maternal deaths in Ghana. The findings of this study will contribute towards spearheading Ghana’s achievement of SDG goal three, target one by 2030 which aims to reduce the global maternal mortality ratio to less than 70 per 100 000 live births [28].

### 1.1 Study aims

This study aimed to investigate stakeholder perspectives to provide a comprehensive understanding of the broader context in which the Ghanaian midwife operates. By comprehensively examining the multiple factors influencing preeclampsia management, we can enhance treatment outcomes and identify key areas that require urgent attention. Using the social-ecological model (SEM) [29] to frame the findings, this study explores, describes, and categorises the influencing factors to midwives’ care of women with preeclampsia in a large tertiary hospital in the capital, Accra.

### 1.2 Socioecological model

The study is based on the Socioecological Model (SEM) [29] a comprehensive model which posits that intertwined relationships exist in healthcare systems thus, the model moves beyond the influence of a single-level factor and considers the interaction of multiple elements within the social environment to provide a deeper understanding of a phenomenon [30, 31]. Initially developed for health promotion and prevention programs, the model has also been used to study various nursing and midwifery topics and has utility in healthcare practice contexts [30, 32]. The SEM stratifies influences to behavior into four levels with interdependent relationships and different levels of impact [29, 33]. At the core, individual-level factors include attributes such as the midwife’s knowledge, attitudes, skills, and perceptions. The second level, interpersonal, encompasses relational factors like interprofessional collaboration and the social relationships between the midwife and the woman experiencing complications [34]. The third level focuses on institutional characteristics, structures, policies, and norms. At the public policy level, the outermost layer, relevant laws, and maternal health policies from local and national sources such as referral policies are included [33]. Therefore, when considering the model, the combined impact of different factors at each level can either support or impede midwives’ management practices.

## 2. Materials and methods

### 2.1 Design

Qualitative descriptive studies aim to explore a naturalistic view of a phenomenon with codes generated from the data to unearth the what and where of events or experiences [35]. As little is known about the underlying factors to midwives’ management of preeclampsia, a qualitative description was relevant to study this phenomenon in its natural setting. In addition, this approach allowed participants to describe their experiences and perceptions. [35]. The study adhered to the consolidated criteria for reporting qualitative research (COREQ) [36] (S1 Appendix).

### 2.2 Participants and sampling

The study used a purposeful sample of stakeholders, including managers and practicing midwives who met specific criteria and provided valuable insights on contextual factors. Managers were selected based on their expertise and leadership roles, with a total of seven participating. Managers were contacted via telephone and email, with consent confirmed through their response. A convenience sample of 35 midwives willingly participated, recruited through printed advertisements and the assistance of a gatekeeper. Eligibility was assessed, and recruitment occurred after obtaining signed informed consent.

Inclusion criteria: Registered midwives, employed within the XXX hospital, working in all maternity areas.

Exclusion criteria: Auxiliary midwives, general nurses working at the department, and midwifery students

### 2.3 Setting

Hospital X is the largest tertiary hospital in Ghana, with a busy maternity department. The department serves regular antenatal attendees and referrals from across the country. With 320 midwives and 70 doctors, the department handles approximately 100 antenatal visits daily and records 10,000–12,000, annual births. High-risk obstetric conditions such as preeclampsia are common among referred pregnant women.

### 2.4 Data collection

Data were collected within three months in 2021 via one-on-one in-depth semi-structured interviews. Due to Covid-19 travel restrictions, all interviews took place via WhatsApp calls which provided secure and encrypted communication [37]. All participants received a plain language statement explaining the study before they provided voluntary informed consent. Managers were asked an opening question about priority issues in preeclampsia/eclampsia management, followed by probing questions. Midwife interviews used a semi-structured guide (S2 Appendix), with open and closed-ended questions. Interviews lasted 30–40 minutes, were audio-recorded, and participants were assigned pseudonyms for confidentiality. Data collection ended after 42 interviews when no additional barriers and facilitators were identified. All participants received mobile airtime as compensation for the internet data used during the interviews.

### 2.5 Data analysis

Braun and Clarke’s six-step thematic analysis procedure was employed due to its suitability for various qualitative data types [38]. The analysis approach was inductive, with coded categories derived directly from the data. After transcribing the interviews, the first author thoroughly reviewed the transcripts and coded the data in conjunction with the second and third authors. Initial codes were derived from interesting features in participants’ narratives and were iteratively grouped based on similarities. Categories were named through discussions and summaries were created by the first author and verified by the second and third authors. Some categories were combined, and subthemes were examined for coherence and theoretical relevance. Compelling narratives that aligned with the research question were extracted from the data set. The socioecological model was used to structure the findings. NVivo (V.11.2.2 QSR International) was used to manage the data.

### 2.6 Ethical approval

This study forms part of a doctoral research project, and ethical approval for the broader project has been granted by Charles Darwin University Human Research Ethics Committee and Korle Bu Teaching Hospital Institutional Review Board and Scientific Committee (CDU-HREC H20118; KBTH-STC/IRB/00013/2021).

## 3. Results

### 3.1 Participant characteristics

In total, the study included 42 participants of which 35 were midwives and seven managers. There were three midwifery heads of service, whilst the other four managers consisted of the obstetric team lead, the chief pharmacist, the head of the laboratory services, and head of the accounts department. Managers clinical work experience averaged 15 years however their managerial experience ranged between 1–4 years. The background characteristics of study participants (midwives) are presented in Table 1.

**Table 1:** Background characteristics of study participants (Midwives).

Variable	Category	N = (35) (%)
**Years of experience**	Less than 5 years	9 (25.7)
	5–10 Years	12 (34.3)
	Above 10 years	14 (40)
**Place of work**	Obstetric emergency/antenatal unit	12 (34.3)
	Labor ward	14 (40)
	Antenatal/postnatal ward	8 (22.9)
	Obstetric recovery	1 (2.8)
**Professional level**	Staff midwife	6 (17.2)
	Senior staff midwife	11 (31.4)
	Midwifery officer	9 (25.7)
	Senior midwifery officer	9 (25.7)
**Highest Educational qualification**	Diploma	15 (42.9)
	Bachelors/master’s degree	20 (57.1)

### 3.2 Themes and subthemes

**Fig 1:**
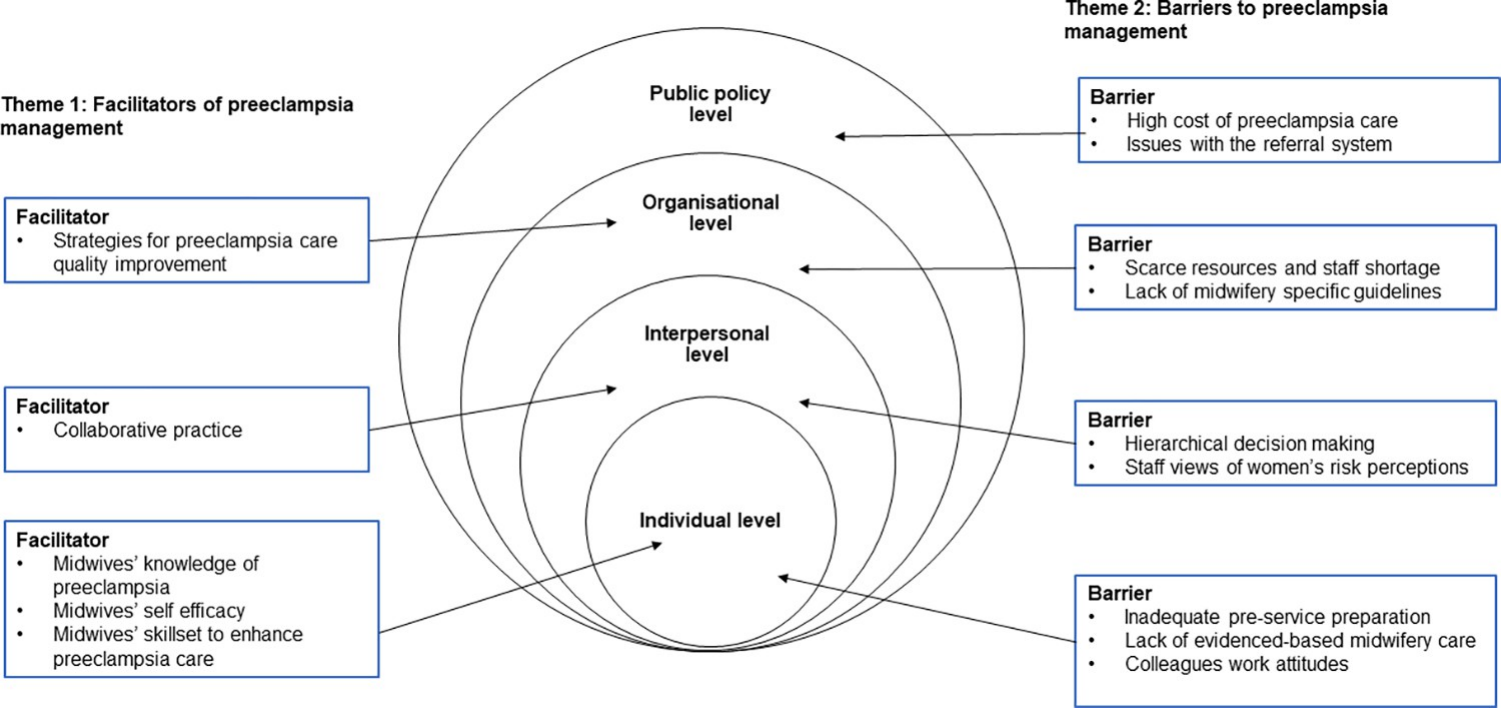
Socioecological model of the facilitators and barriers to midwives’ management of preeclampsia.

Two major themes were identified: 1) facilitators of preeclampsia management and 2) barriers to preeclampsia management. Each theme had corresponding subthemes which were organised within the socioecological model (Fig 1).

#### 3.2.1 Facilitators of preeclampsia management

*3*.*2*.*1a Individual level facilitators*. Three facilitators were identified at the individual level and included midwives’ knowledge of preeclampsia; midwives’ self-efficacy; and midwives’ skillset to enhance preeclampsia care.

### Midwives’ knowledge of preeclampsia

Midwives had knowledge and understanding of preeclampsia acquired from clinical experience. They discussed the diverse clinical presentation of preeclampsia emphasising how the recognition of subtle symptoms could indicate a rapid decline in the woman’s condition. They correctly identified key diagnostic elements such as the blood pressure parameters, and important clinical assessments, and they knew the associated risks including HELLP syndrome and eclampsia. Clinical protocols helped improve their knowledge.

“*Occasionally the women come in healthy……*. *It is after you check the blood pressure and the urine proteins that you get to know that there is a problem*. *The BPs are elevated*, *like above 140/90mmHg and there is proteinuria*. *They have oedematous feet*, *frontal headaches*, *and other symptoms”* MW8

“*The protocols serve as a reference point…*… *It has really helped me know what to do”* MW18.

### Midwives’ self-efficacy

Midwives expressed that they had mastery of preeclampsia management gained from hands-on midwifery practice, enabling them to safely provide lifesaving interventions. Midwifery managers shared their trust in the midwives’ skills and professional competencies.

A midwifery manager said: *“I must say our midwives are very competent*. *We get a lot of cases*, *midwives know what to do and are all hands-on deck”* SM3

A senior midwife shared: *“I have been at the antenatal unit for eight years…*.. *If I am to score myself on emergency management*, *I would give myself 8 [on a scale of 1–10]”* MW35

Junior colleagues replicated the clinical decision-making style of seasoned midwives they perceived had more self-efficacy: “*Midwives who have been in the system for a long time have rich experience*. *I learn from them and manage cases the way they do”* MW20

### Midwives’ skillset to enhance preeclampsia care

Having the right skills is necessary to prevent further complications. Midwives discussed the vital skills they possess and shared their experiences mentoring new graduates to acquire those same skills. A midwife describes the care of a woman experiencing an eclamptic seizure:

“*When we receive her*, *we recheck the BP and the urine*. *After confirming the BP*, *we come in and start with the drug protocols*. *We insert a urinary catheter before giving magnesium sulfate so that if anything untoward is happening*, *we can confirm from the urine output”* MW6.

They recounted reflecting in action and teaching skills to colleagues:

“*I usually try to think of the most urgent problems the woman presents*. *Over time I know what to do and act promptly”* MW33

Newly employed graduates are taken through a 12-month rotational mentoring program to enhance a smooth transition at the unit. The chief midwifery officer explains:

*“Recently we had a lot of midwives employed*, *that is*, *from April*. *When they come*, *we pair them with a senior colleague at their unit*. *The senior one oversees what the junior is doing”* SM1

One midwife recounts the usefulness of clinical mentoring:

“*When I came here as a staff midwife six years ago*, *I was paired with a midwifery officer*. *I could tackle preeclampsia on my own after the two-month period I spent at the uni*t” MW7

*3*.*2*.*1b Interpersonal level facilitators*. One facilitator was identified at this level. The subtheme of collaborative practice was centered around teamwork and team-based learning.

### Collaborative practice

Participants emphasised the significance of collaborative practice to effect positive change. They described collaborative working between midwives and obstetricians highlighting the benefits of interprofessional learning and teamwork.

One midwife explains: *“What works well is the doctors and midwives working hand in hand*. *A doctor may say let’s give Labetalol instead of Magnesium sulphate and a midwife may also suggest adding it because of the high BP……So they discuss…*. *the two parties agree*, *draw a conclusion and do it”* MW6

Interprofessional team-based learning took the form of a daily clinical audit: “*Basically at 8 am every morning we meet*, *and cases managed throughout the past 24 hours are presented*. *If there were some lapses*, *we discuss those*, *then we all take a cue from that mistake so that it is not repeated*” SM1

*3*.*2*.*1c Organisational level facilitators*. One facilitator was identified at this level. This subtheme refers to organisational quality improvement strategies aimed at better preeclampsia care outcomes.

### Strategies for preeclampsia care quality improvement

Participants highlighted proactive measures as part of the hospital’s response to preeclampsia management such as visible clinical protocols, streamlined referral processes with a liaison referral team, and an educational support program to improve women’s understanding of pregnancy-related complications. Participants also discussed the department’s effective recognition and response system for preeclampsia cases. Staff dedication and an understanding of the importance of timely and accurate assessments were crucial in reducing adverse outcomes.

“*We have a protocol pasted all around so that in case you have even forgotten*, *or you are new to the system*, *it is there to help or guide you*.” MW10

*“We have a referral team in the department*. *If they [another hospital] want to refer a case*, *they will call*, *and we would tell them what to do*. *Previously on weekends*, *this place would be flooded with cases”* SM3

Midwives emphasised the significance of triage for timely interventions:

“*For referrals*, *midwives quickly do the registration to capture the demographic data*. *Then we go through the referral letter…*. *Depending on the BP range*, *she is quickly arranged to be seen and admitted*”SM1

#### 3.2.2 Barriers to preeclampsia management

Nine barriers were identified across all four levels of the SEM.

*3*.*2*.*2a Individual level barriers*. The barriers were: Inadequate pre-service preparation; lack of evidence-based midwifery care; and colleagues’ work attitudes.

### Inadequate pre-service preparation

The midwives reached a consensus that their pre-registration midwifery education lacked practical relevance, especially in addressing pregnancy complications such as preeclampsia. They highlighted the imbalance between theoretical learning and clinical placements needed to develop confidence and competence. Midwives expressed frustration over their inability to apply the theoretical knowledge they had obtained in the actual practice environment.

“*No*, *not at all*! *I don’t think it was adequate*. *They taught us*, *but we didn’t see the practicality of it until we experienced it in the field*” MW1

Another added: *“At the diploma level there are few practical sessions on the management of abnormalities in pregnancy*” MW27.

### Lack of evidence-based midwifery care

Midwives described clinical management practices for which there is no scientific basis, including routine weight checking, dietary sodium restrictions, bed rest, and exercise. However, some midwives acknowledged the inefficiency and ritualistic nature of these interventions.

“*We know diet and exercise play an important role in managing hypertension cases*, *so we want them to cut down their salt levels*. “MW18

One midwife expresses her desire to be abreast with current management recommendations:

*“I know knowledge is not static…*., *so I try as much as possible to keep up with new things and new ways of management”* MW34.

### Colleagues’ work attitudes

Midwives raised concerns about the work ethic of some colleagues. They perceived that some were not suited to their work and showed negative attitudes which could compromise the quality of care. Participants cited examples where colleague midwives were not prepared and failed to identify clinical deterioration:

“*Our work has a lot to do with observation*, *you have to pay attention to details*. *For example*, *you tell a midwife that when someone’s blood pressure reads above 160/110mmHg*, *it should be documented and reported*. *Yet this midwife will record a high value and move on”* MW15

The midwifery management was aware these negative attitudes. In response one said:

*“There is more room for improvement because our management as midwives will help the obstetricians manage the women very well to prevent most of the damaging things that happen”* SM4

*3*.*2*.*2b Interpersonal level barriers*. **Hierarchical decision making.** Midwives discussed how institutional hierarchies restricted their role in managing preeclampsia. Due to their traditionally lower status than their medical counterparts, they lacked professional autonomy and felt obliged to be dutiful subordinates to obstetricians. Consequently, they felt stifled and were hesitant of shouldering responsibility without the physician’s orders.

*“Most midwives are not given the room to perform*. *We do not go out of our way to practice because of the fear that if something goes wrong*, *we will be held responsible*.*”* MW24

One midwife shares her frustration:

*“From the symptoms she was exhibiting*, *I suspected she had preeclampsia and suggested it to my team head*, *a doctor*, *but he ignored me……*… *Later*, *the woman started fitting in our presence”* MW13

### Staff views of women’s risk perceptions

Participants noted that women were unaware of the potential danger of preeclampsia, leading to poor decision-making. Instances of skepticism, care refusal, and worsened readmissions were shared. They attributed women’s actions to a lack of knowledge of preeclampsia and sociocultural beliefs:

*“There was this woman who was referred from a polyclinic with preeclampsia…*.*she refused admission*. *The next day*, *unfortunately*, *her relatives came with a dead body*. *Probably if she had known something about her condition*, *she wouldn’t have refused the admission”* MW3

Another added: *“Some women decide to go and see their pastor or some herbalist somewhere*. *Others too say*: *my husband is not at home……*.*they have to inform their husbands”* MW26

*3*.*2*.*2c Organisational level barriers*. **Scarce resources.** Participants highlighted the challenges faced due to scant resources and a shortage of personnel which compromised the quality of care. Due toCovid-19, competing priorities meant that the situation had worsened, and supplies such as urine dipsticks were in short supply.

*“The hospital does not give enough consumables for work*. *Let’s take the urine dipstick for instance*. *It’s just a few and you have about 80 to 100 women per day*. *Because they are few*, *we cut the strips into pieces so that at least we will have one for each woman*. *The BP apparatus are also few*, *most are faulty”* MW35

Some problems have been in existence pre-pandemic. The absence of an intensive care unit and a functioning laboratory in the department is usually a cause of unusual delays.

“*We don’t have machines for analysing the blood chemistries and lipid profiles*. *The liver function tests*, *the clotting profile*, *and full blood count have to be taken to the central lab for analysis*.*”* SM6

Another manager added:

*“We don’t have an ICU; we use the main ICU of the hospital*. *There is no allocation of beds for O& G*. *It depends on the availability and who comes first”* SM5

Some participants also complained about staffing:

*“Shortage of staff has always been the headache of the unit*. *As I mentioned earlier*, *we can have about 20 cases with three midwives*. *How well do you think the care that will be rendered will be*? *Will it be up to the level that we expect*? *No*!*”* MW24

One participant shared how balancing high-dependency care with routine care labor care often led to the failure to complete interventions:

“*Sometimes it becomes so hectic that the blood pressure management for the woman doesn’t go on well*. *We need to administer magnesium sulphate every four hours*, *but it ends up becoming every six hours or seven hours…*.*” MW21*

Participants’ descriptions highlighted a lack of institutional commitment as midwives were financially responsible for any personal development they embarked on:

*“They organise workshops that midwives pay to attend*. *I have to pay for the workshop and that even discourages me*. *Since I came here*, *I have gone to those workshops just once*. *I needed the certificate to renew my pin which is why I attended”* MW31

### Lack of midwifery-specific guidelines

Midwives identified a lack of specific preeclampsia protocols. Whilst they found the protocols helpful and followed them, they expressed concern that the recommendations were not explicit, fearing potential mismanagement and consequences for the women involved.

*“The protocol…*… *It’s mostly jammed together; admit the woman*, *give that*, *etc*. *It’s like every staff category should know what they are supposed to do*… .. *But it’s not specified*, *like underlined*, *that midwives do this and doctors to do other tasks”* MW5

*3*.*2*.*2d Public policy barriers*. Two barriers were identified at this level: the high cost of preeclampsia care and issues with the referral system.

### The high cost of preeclampsia care

Women admitted with a diagnosis of preeclampsia have huge bills including the cost of drugs, laboratory tests, and overall expenses. Maternity hospitalisations for pregnancy cause a further financial strain as women are unable to pay the bill.

“*Money is a major problem*. *Sometimes when they come [women]*, *and we request laboratory investigations*, *they don’t have the money to do it*. *You know the health insurance doesn’t cover all the things”* MW26

*The chief pharmacist explains*: *"The national health insurance is supposed to take care of some of the drugs which have been one of my waterloos since I came in 2017…*..*the woman will have to go across the road to get their medications”* SM2

Participants described the dire situation where bills were sometimes written off by the social welfare department and other cases where staff used their money to cater for the woman’s bills.

*“One thing that we do is if a patient [woman] can’t afford her medications if staff can afford to*, *we buy it for them”* MW5

### Barrier: Issues with the referral system

Early detection and prompt referral to a higher level of care are crucial for preeclampsia outcomes. Participants noted discrepancies in the referral system including omissions in first-line treatment, a lack of uniform referral criteria and women being unnecessarily held up at lower levels before being referred:

“*Some hospitals delay unnecessarily*. *Sometimes they keep her knowing very well that her blood pressure is very high meanwhile the best way is to refer her to a higher institution*.*”* MW3

The national ambulance service provides emergency transport to referral hospitals in Ghana. Yet they are few, slow, ill-equipped, and unreliable. Participants voiced their concerns:

“*All they [referring to midwives in other lower-level hospitals] have been told is if the patient [woman] is having high blood pressure with proteinuria of this*, *refer her to X*. *Some midwives come with referrals in taxis and when we ask why they say*: *oh no ambulance”* MW12

## 4. Discussion

This qualitative research explored and described the factors influencing midwives’ management of preeclampsia in a Ghanaian tertiary hospital within a socioecological framework. Our study found that there are distinct multi-level facilitators and barriers to midwives’ preeclampsia management. Our findings are consistent with the growing body of literature examining the quality of care in preeclampsia and eclampsia management in Ghana [15, 16, 27]. Common barriers evident from those studies included a shortage of personnel, poor attitudes, lack of hospital infrastructure for preeclampsia care, cost, and women’s inherent lack of knowledge and awareness of preeclampsia. Additionally, this study expands the existing literature by identifying facilitators that can enhance midwives’ effectiveness at preeclampsia management.

Skilled and knowledgeable midwives are essential for high-quality preeclampsia treatment [39]. Our findings indicate that midwives in tertiary care centers play a central role, requiring competence, confidence, and on-the-job training to provide effective care and support. These findings provide support for improving Ghanaian midwives’ competencies through investments in formal education and ongoing training to optimise midwives’ impact in preeclampsia care [15, 40]. More research on individual-level determinants and interdisciplinary care is needed to guide for implementing changes in maternity care systems. Midwifery leadership in Ghana should advocate for more autonomous midwifery practice to promote continuity and reduce fragmented care in preeclampsia across the health system.

One of the key issues that emerge from these findings is the influence of negative risk perceptions on pregnant women’s health-seeking behavior. Social, cultural, and religious determinants contribute to skepticism and non-compliance with health information and suggested behavior modification [16, 41, 42]. Lack of local terminology for preeclampsia leads to confusion, [42, 43], self-medication, and hospital care avoidance. To address this empowering women and families is essential. Public health awareness programs must be prioritised to facilitate early detection and prompt management of preeclampsia. Expanding educational programs and conducting further research on community perceptions is crucial with the involvement of international organisations like local chapters of Action on Preeclampsia and the Preeclampsia Foundation [44]

Relatively little literature can be found on the facilitators of preeclampsia care at the health organisation level, especially in Ghana. The study findings contribute to existing literature [16, 45, 46] assessing the institutional capacity and preparedness to provide adequate preeclampsia care. Despite the hospital’s commitments to quality care, tangible benefits for women have not yet been achieved [47]. Costs, logistics, and referrals remain a challenge to women’s access to care and are counterproductive in maternal morbidity and mortality [16, 48]. These barriers align with those faced by other LMICs [45]. Stakeholders must therefore prioritise the equitable distribution of maternal healthcare resources. Ghana’s existing initiatives have had limited success and sustained investment is required to accelerate solutions to the barriers identified in this study and also discussed in similar contexts [46].

The costs associated with preeclampsia far outweigh the minimum wage in Ghana which is currently $1.70 per day. While the Ghana National Health Insurance Scheme (NHIS) subsidises 90% of the cost, registered women pay $109 and those unregistered pay $184., with the cost increasing when women have a caesarean birth. Previous studies in the United States of America and Ireland have shown substantially higher costs associated with preeclampsia care [49, 50]. This highlights the need for expanded maternal healthcare financing to address the significant financial strain on families and the health system. Overall, effective midwifery care outcomes rely on a functioning health system that addresses access, affordability, availability, and appropriateness.

### 4.1 Limitations

A major limitation was conducting online interviews due to covid travel restrictions. This method restricted our ability to observe the nonverbal cues including body language. The bandwidth, lighting, and the participant’s video quality also limited what was visible and may have impacted data quality [51]. Tailored instructions and troubleshooting tips were sent with individual invitations with the option of switching to audio calls. Also, this study does not consider the women’s voices and realities as these were not the focus of the investigation.

## 5. Conclusions

We have identified several multi-level facilitators and barriers to preeclampsia management. The identified facilitators can be leveraged to develop and implement quality improvement initiatives to enhance midwives’ practice whilst urgent attention is focused on eliminating the system-wide barriers beyond the midwife’s control to ensure better outcomes in preeclampsia. Given the role of midwives in the continuum of women’s care, there is substantial potential for midwives to impact maternal outcomes of preeclampsia if they are well supported, have opportunities for specialised training to improve their competency, and can perform the whole range of functions to their full professional scope.

## Supporting information

S1 Appendix Consolidated criteria for reporting qualitative studies (COREQ): 32-item checklist. (DOCX)

S2 Appendix In-depth interview manual (Midwives). (DOCX)
