# Supplementary material for: A socioecological description of the influencing factors to midwives’ management of preeclampsia in a Ghanaian tertiary hospital
Source: PLoS One. 2023 Sep 13;18(9):e0291036. doi: 10.1371/journal.pone.0291036 (PMC10499208; doi:10.1371/journal.pone.0291036)
Supplement: S2 Appendix — (DOCX) [file pone.0291036.s002.docx]

**S2 Appendix: In-depth interview manual (Midwives)**

Project title: Exploration of factors influencing midwifery management of preeclampsia/eclampsia in a Ghanaian teaching hospital

**Introduction**

This interview explores the factors influencing midwifery management of preeclampsia/eclampsia in a Ghanaian teaching hospital. The study has been approved by the Charles Darwin University human research ethics committee (CDU-HREC) and the Korle-Bu Teaching Hospital (KBTH) ethical review board. Your participation in this research is completely voluntary. Confidentiality will be ensured as limited identifying information will be collected and this information will be de-identified and not shared. I would like to remind you to sign a consent form if you have not already done so. One copy of the informed consent form will be given to you and a second copy will be kept by the interviewer. I would also like to remind you that the interviews will be digitally recorded for later transcription. The transcriptions will be de-identified, stored, and will later be transferred to a password secured computer. Finally, you have the right to withdraw from an interview at any time without any repercussions. Thank you for taking part in this study. Do you have any questions before we begin?

**Warm up and background information**

It is a pleasure to meet you. I bring you greetings from my supervisors and colleagues. I am also a midwife and find it helpful to engage with other midwives and find out about their work. First, I’d like you to introduce yourself. Please select a pseudonym and tell me about years worked, educational qualifications, and professional rank.

Years worked……………………………………………

Educational qualifications………………………….

Professional Rank……………………………………….

Midwifery role…………………………………………..

I am just going to give you a couple of minutes to think about your personal or your organisation’s experience of caring for women who develop preeclampsia and eclampsia.

**Introductory question**

1. What do midwives normally do for women diagnosed with preeclampsia and eclampsia?

*Probes: What do you know about preeclampsia? Can you tell me some of the things that you do?*

*What is expected of you regarding a woman who comes for routine care versus a woman who has been referred on account of preeclampsia?* *let’s say a woman begins to have a seizure whilst you’re reading her referral notes, how would you handle that?*

*What skills do you need to detect and care for the woman with preeclampsia?*

*Do these skills cut across all the maternity units? Unpleasant experiences?*

1. Can you tell me about any protocols or guides used to aid the management of preeclampsia and eclampsia?

*Probes: What preeclampsia management guidelines are available to midwives? What aspects of it are useful? How could these guidelines or protocols be improved? Are you aware of any recent happenings or changes in preeclampsia management guidelines?*

1. How do you feel about your own and other midwives’ competence in the management of preeclampsia and eclampsia?

*Probes: How confident are you that you can care for a woman with preeclampsia or eclampsia? Are there any skills you feel you need to improve?*

*Do you think your colleagues have the right knowledge and skills to manage preeclampsia cases?*

*Have you noticed any differences between how senior colleagues care for women vs the new or younger midwives?*

1. At the point of registration do you think midwifery pre-service training prepares midwives to adequately to manage preeclampsia/eclampsia?

*Probes: What did you think of the training? Were the resources and course materials useful? What practical training specific to preeclampsia management can you recall? From your point of view what other problems exist at the training school level? Is it different at the degree vs diploma level?*

1. What do you think works well in the management of preeclampsia and eclampsia in this hospital?

*Probes: Other than midwives, who are the other professionals involved in the management of preeclampsia and eclampsia and how do you think these group of people work as a team? How important is the midwife’s role in the management team? Do midwives support each other in acquiring more knowledge and sharpening skills needed for preeclampsia management? In your view, are there in-service training activities targeted at preeclampsia specifically?*

1. What are some of the challenges that midwives face as they care for women with preeclampsia and eclampsia in the hospital and in your unit?

*Probes: Can you talk about some hard situations you have faced whilst performing your everyday work? Do you think there are any hinderances in the care of women diagnosed with preeclampsia? Or any limitations as to what the midwife can or cannot do? Do you think you have enough resources at your unit? How do you cope with the problems you have just listed?*

1. How do you think the management of preeclampsia and eclampsia can be improved?

*Probes: Which improvements do you think are necessary at the moment? What might assist midwives in particular to provide high quality care?*

**Concluding question**

- What is the most important thing to you out of all the issues we discussed today?

**End of interview**

Thank you for participating.

This has been a very productive interview.

I’d like to remind you that names of the hospital and participants will be de identified, and we will only use quotes and themes
